# Enhanced recovery protocol for congenital duodenal obstruction – initial experiences with development and implementation

**DOI:** 10.1007/s00383-024-05951-2

**Published:** 2024-12-27

**Authors:** Henrik Røkkum, Martin Alavi Treider, Wenche Bakken Børke, Janicke Bergersen, Kristoffer Lassen, Ragnhild Støen, Thorstein Sæter, Kristin Bjørnland

**Affiliations:** 1 Department of Pediatric Surgery, Oslo University Hospital, Nydalen, P. O. Box 4950, N-0424 Oslo, Norway; 2 Institute of Clinical Medicine, University of Oslo, Oslo, Norway; 3 Department of Anesthesiology, Oslo University Hospital, Oslo, Norway; 4 Children’s Surgical Department, Oslo University Hospital, Oslo, Norway; 5 Department of Hepato-Pancreato-Biliary (HPB) Surgery, Oslo University Hospital, Oslo, Norway; 6 Institute of Clinical Medicine, UiT, the Arctic University of Norway, Tromsø, Norway; 7 Department of Clinical and Molecular Medicine, Norwegian University of Science and Technology, Trondheim, Norway; 8 Department of Neonatology, St. Olav’s Hospital, Trondheim University Hospital, Trondheim, Norway; 9 Department of Pediatric Surgery, St. Olav’s Hospital, Trondheim University Hospital, Trondheim, Norway

**Keywords:** Enhanced recovery after surgery (ERAS), Enhanced recovery protocol (ERP), Congenital duodenal obstruction, Duodenal atresia, Neonatal surgery, Postoperative complication

## Abstract

**Background:**

The experience with Enhanced Recovery After Surgery^®^ (ERAS^®^) protocols in neonatal intestinal surgery is very limited. We present the development and implementation of an Enhanced Recovery Protocol (ERP) designed specifically for neonates treated for congenital duodenal obstruction (CDO), and early outcome after implementation.

**Methods:**

An ERP for CDO was developed and implemented. Experiences with ERP development and implementation are described. Early clinical outcome in patients treated before (January 2015–Descember 2020) and after (February 2022–September 2024) implementation were compared. Ethical approval was obtained.

**Results:**

A multidisciplinary ERP team was established. The ERP for CDO was developed with stakeholder involvement. Implementation was challenging, but with close follow-up and frequent meetings with the involved medical disciplines, an overall ERP compliance of 80% was achieved for the 21 patients treated after implementation. Compared to 40 patients treated before ERP implementation (January 2015-Descember 2020), the use of minimally invasive surgery increased and time to first postoperative enteral and breast feed were reduced, without increasing the rate of postoperative complications.

**Conclusions:**

This study presents an ERP specifically designed for CDO with a unique description of our experiences with the development and implementation process. Early results suggest that this ERP for CDO is feasible and safe.

## Background

Enhanced Recovery After Surgery^®^ (ERAS^®^) protocols are characterized by standardization of treatment through multimodal evidence-based routines covering the whole perioperative course [1]. The benefits of ERAS protocols, often termed Enhanced recovery protocols (ERPs) have been well documented in adult surgery and include reduced complication rate, earlier return of gastrointestinal function, shorter postoperative hospital stay, and reduced total hospital costs [2]. Due to these advantages, ERPs have increasingly been applied and is now standard of care in most adult surgical specialties [3].

Implementation of ERAS/ERPs has been slow in pediatric surgery. This is not unexpected, since pediatric surgery is characterized by a range of many low-volume and rare conditions. Additionally, the evidence for many parts of the perioperative management is weak. However, in recent years, ERPs have been demonstrated to be feasible and beneficial in several fields of pediatric surgery, including urology, gastrointestinal, and thoracic surgery [4–8]. Reduced time to postoperative enteral feeding, reduced postoperative opioid use, and shorter hospital stay are among the reported benefits.

In neonatal intestinal surgery, the experience with ERAS is still very limited. The first ERAS consensus guidelines for neonatal intestinal surgery were published in 2020 and were recently updated to apply to all surgical neonates [9, 10]. Aiming to encompass neonates with a wide range of diagnoses and procedures, these ERAS guidelines are general and not designed to address specific diagnostic entities. Some of the recommendations, such as refeeding via an enterocutaneous fistula, will not always be relevant or applicable, and certain diagnoses may require more diagnose-specific recommendations, such as those concerning postoperative enteral nutrition.

With an incidence of 1–3 cases per 10,000 live births, congenital duodenal obstruction (CDO) repair is one of the most commonly performed gastrointestinal procedures in neonates [11, 12]. Despite being a regularly performed operation, there is no consensus regarding the handling of perioperative challenges unique to CDO, and practices vary considerably between institutions [11]. A recent evaluation of the management of CDO in Norway revealed almost no change in treatment or improvement in outcome during the last decades [13]. Three publications describe the use of ERP for CDO [14–16]. However, a dedicated ERP for the perioperative management of CDO based on the ERAS guidelines for neonatal intestinal surgery has not been published. We have, therefore, updated and standardized the management of CDO through an ERP based on the best available evidence and the ERAS guidelines for neonatal intestinal surgery. The aims of this study were to present our ERP, experiences with development and implementation, and early results after implementation.

## Methods

### Study design and context

This is a two-center combined implementation and clinical outcome study. After protocol development, the ERP for CDO was first implemented at Oslo University Hospital (site 1) in February 2022, and an executive decision to treat all eligible patients according to the protocol was made. After the initial phase of implementation at site 1, the ERP was implemented at St. Olav’s Hospital (site 2) in January 2023. In Norway, treatment of CDO is centralized to these two hospitals. Hence, this is a nationwide study.

In patients treated after ERP implementation, protocol compliance and clinical outcome were registered prospectively after obtained written consent. Compliance with the recommendations in the protocol was determined as treatment in line with the definition for the respective recommendations. The authors’ experiences with implementation of the ERP are anecdotally described. Reporting recommendations from the RECOvER checklist was followed when applicable [17]. In patients treated before ERP implementation, clinical outcome were registered through chart review. Fluid balance 24 h from surgery was calculated as the difference between intravenous input and urinary output the first 24 h after induction of anesthesia. Length of hospital stay was defined as hospital stay at site 1 or 2, and hospital stay in patients transferred to a community hospital before discharge to home was not registered.

### Pre-protocol routines

Before ERP implementation, the perioperative care of patients with CDO was mainly at the discretion of the treating surgeon. Verbal parental information was provided, but standardized written information was not available. Standard surgical access was through an upper transverse laparotomy. The pediatric anesthesiologists have continually updated their practice towards a more fluid restrictive approach to avoid fluid overload, and perioperative hypothermia is prevented by pre-warming the operating room, use of heated operating table, underbody forced-air warmers, and continuous intraoperative temperature monitoring. Postoperative pain was evaluated with COMFORT scale and face, legs, activity, cry, and consolability (FLACC) pain scale [18, 19]. Analgesic medication typically included regularly administered acetaminophen and opioids as needed, with opioids sometimes administered as continuous infusion. Before ERP implementation, no formal protocol for postoperative feeding or aspiration of gastric residuals was used. Postoperative feeding was guided by the volume and color of gastric residuals and based on the surgeons’ preferences. Generally, only small amounts of gastric residuals were accepted, and enteral feeding was delayed until a reduction in gastric residuals was observed. Lingual sucrose was frequently used to soothe the patient. Transanastomotic feeding tube (TAFT) was used sporadically [20]. Parenteral nutrition was administered until enteral feeding at a minimum of 100 ml/kg/day had been established.

### Protocol development

A multidisciplinary ERP team including pediatric surgeons, a pediatric anesthesiologist, and a pediatric surgical nurse was first established at site 1. A gastrointestinal surgeon with broad experience in development and implementation of ERAS in adult surgery had a mentoring role throughout the study.

Several meetings with all the involved medical disciplines at site 1 were arranged. Herein, challenges in the perioperative care of neonates with CDO were identified, the best available evidence regarding treatment of patients with CDO were discussed, and principles of ERAS and the planned implementation of ERP for CDO were presented. To ensure involvement of the parents in the ERP development process, parental experiences and needs were assessed through a focus group discussion with parents of neonates treated for CDO [21]. The parents expressed a considerable need for information. They further reported a feeling of separation from the baby and the need for being more involved in caretaking of the baby postoperatively. This was to a certain degree aggravated by the use of medical tubes and monitoring systems. Furthermore, the mothers had severe concerns regarding pumping and breastfeeding. Therefore, we made a written leaflet with standardized parental information regarding CDO. Minimizing medical equipment, early parental involvement, and early breastfeeding were also included in the protocol. Throughout this process, with stakeholder involvement and based on the ERAS guidelines for neonatal intestinal surgery, an ERP for CDO was developed (Table 1) [9]. Recommendations in the ERP that particularly differed from previous practice, included surgical practice and postoperative feeding. Subsequently, an ERP team consisting of a pediatric surgeon and two neonatologists was then established at site 2. They were presented to the ERP, and the implementation process at site 2 was initiated.

**Table 1:** Patient demographics before and after ERP implementation

	Before ERP implementation (2015–2020) *n* = 40	After ERP implementation (2022–2024) *n* = 21	*p*-value
Girls, n (%)	17 (43)	12 (57)	0.277
Gestational age at birth (weeks), mean (SD)	36.9 (2.4)	36.9 (2.1)	0.986
Birth weight (grams), mean (SD)	2668 (640)	2610 (603)	0.734
Prenatal diagnosis, n (%)	22 (55)	16 (76)	0.105
Age at surgery (days), median (IQR)	2 (4)	2 (2)	0.439
Concomitant anomalies, n (%)			
Patients with any concomitant anomaly	18 (45)	7 (33)	
Down syndrome	13 (33)	5 (24)	0.379
Congenital heart disease	8 (20)	2 (10)	
Gastrointestinal anomalies			
Esophageal atresia	2 (5)	1 (5)	
Intestinal malrotation	3 (8)	1 (5)	
Anorectal malformation	1 (3)	1 (5)	
Hirschsprung’s disease	0	1 (5)	
Unknown	3 (8)	0	
Other	3 (8)	0	

Reducing the surgical trauma with minimal invasive surgery is a central concept in ERAS in adult surgery. The level of evidence regarding benefits of laparoscopy in neonatal surgery is weak, and minimal invasive surgery is not included in the ERAS guidelines for neonatal surgery [9, 10, 22]. Laparoscopic CDO repair is feasible, but technically challenging. Compared to laparotomy, laparoscopic CDO repair has been found to reduce time to enteral feeding and the risk for wound infections, and to shorten the hospital stay, without increasing the risk for postoperative complications [23]. Further, long-term patient-reported follow-up studies indicate that the surgical scar from an upper transverse laparotomy in neonates may be cosmetically unfavorable [24, 25]. CDO repair via a supraumbilical mini-laparotomy is feasible, less technically challenging than laparoscopy, and has an excellent cosmetic outcome compared to an upper transverse laparotomy [26, 27]. Hence, we recommend to use either laparoscopic or supraumbilical access for CDO repair in the current ERP, depending on the surgeon’s expertise.

To reduce postoperative opioid use, we recommend wound infiltration with bupivacaine and acetaminophen regularly. Dexmedetomidine until extubation and clonidine as required following extubation should be considered. If adequate analgesia is not achieved, the smallest effective opioid bolus is used. There is no tradition for regional anesthesia in neonates undergoing intestinal surgery in our centers. Due to an empirically very low opioid requirement after CDO repair, regional anesthesia was not included as a recommendation in the current ERP, despite being recommended in the ERAS guidelines for neonatal intestinal surgery [9].

**Fig. 1:**
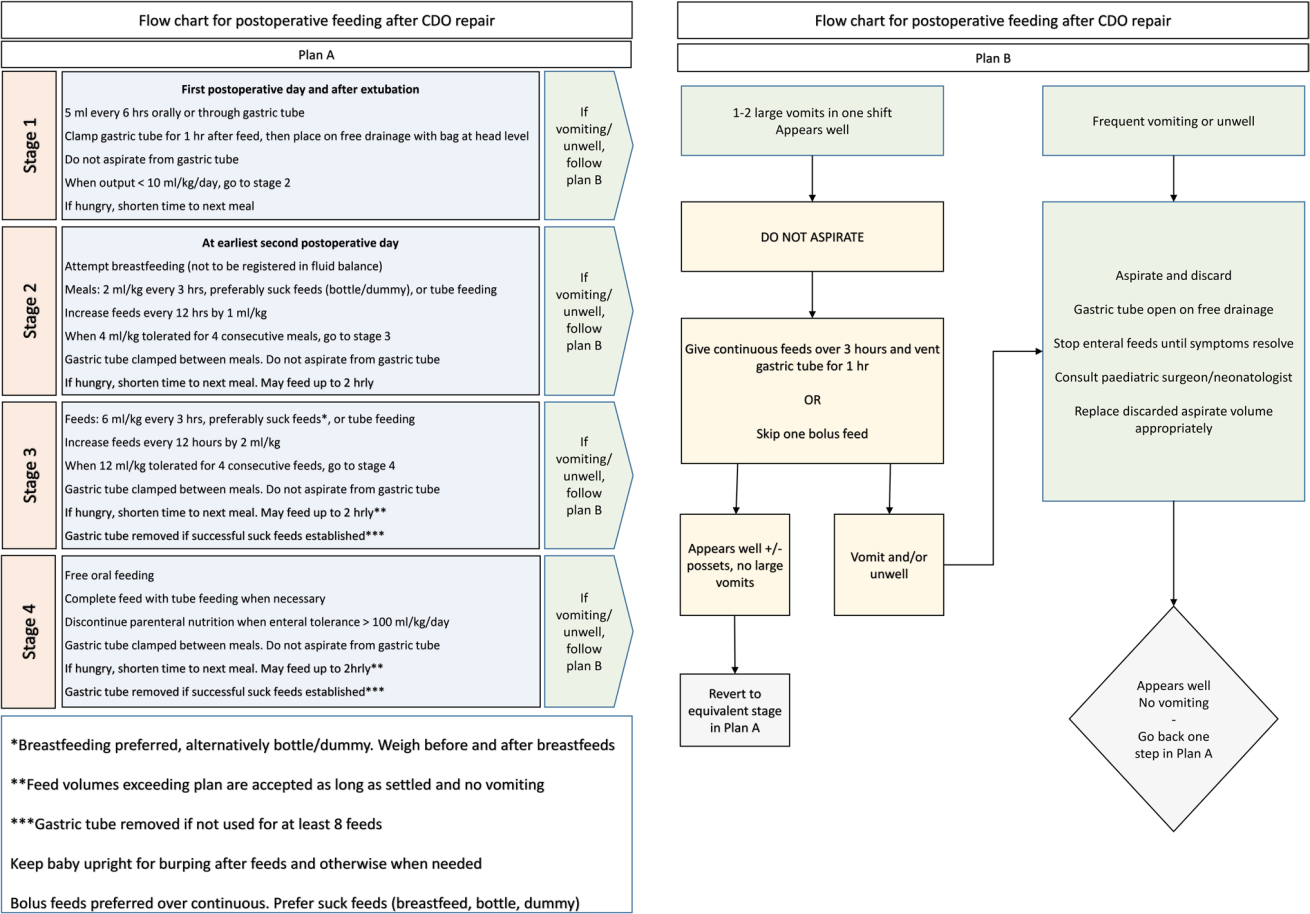
Protocol for postoperative feeding in patients treated for congenital duodenal obstruction (CDO)

The necessity for a postoperative feeding protocol was recognized, and a flow chart for postoperative feeding after CDO repair was composed (Fig. 1). In order to reduce the numbers of drains and tubes, and to facilitate early breast feeding, we decided to not include a TAFT in the feeding protocol. Measures for maintaining normothermia and adequate fluid management and the use of Safe Surgery checklist had already been implemented prior to the development of the ERP for CDO. Hence, this protocol did not involve any change of practice regarding thermoregulation, fluid management, or the use of a perioperative checklist.

### ERP implementation and revision

Prior to ERP implementation, familiarization with the protocol was ensured through separate meetings at both centers between both ERP teams and colleagues in all involved disciplines including pediatric surgeons, neonatologists, pediatric anesthesiologists, and nurses in all involved departments, respectively. After implementation, the perioperative course of patients treated for CDO was monitored closely by the ERP team members. Challenges and uncertainties were addressed, and the protocol was revised as needed.

### Patient population

Neonates treated surgically for CDO within 28 days after birth at site 1 (February 2022–Sepbember 2024) and site 2 (January 2023–September 2024) were treated according to the ERP and included in the study. For comparison of clinical outcome, neonates treated for CDO before ERP implementation at site 1 and 2 from January 2015 to December 2020 underwent chart review. Neonates with gestational age at birth < 32 weeks or with major concomitant anomalies with need for prolonged intubation and neonatal surgical repair were excluded from the study.

### Data analysis

Descriptive statistics are presented. Protocol compliance was calculated as the number of interventions fulfilled out of the applicable recommendations. High compliance for each recommendation was pragmatically defined as > 80% protocol compliance. Categorical variables are presented as frequency and percentage. Numerical variables are presented as mean and standard deviation (SD) for normally distributed data and as median and interquartile range (IQR) for not normally distributed data. Statistical analysis were performed in Stata. Chi-square were used to compare categorical variables. Student t-test and Mann–Whitney U-test were used to analyze normally and non-normally distributed data, respectively.

## Results

Forty-four patients were treated for CDO before ERP implementation (January 2015-Descember 2020). Four patients were excluded due to prematurity (n = 3) and congenital heart disease (*n* = 1). Twenty-one patients were treated for CDO after ERP implementation (February 2022-Sepbember 2024). The groups were demographically comparable (Table 1).

### Compliance with ERP principles after implementation

The overall pooled protocol compliance rate was 80% (215/268). High protocol compliance was found for nine of the 18 recommendations (Table 2). All patients had their first feed within 48 h after surgery, and breastfeeding was the main nutrition at discharge in 90% of the patients. Low compliance (< 80%) was found for four recommendations, in particular related to maintenance of normothermia and to use minimal invasive surgical access. Five recommendations were not possible to evaluate, either due to non-specific phrasing of the recommendation or lack of documentation in medical records.

**Table 2:** Compliance to an Enhanced Recovery Protocol (ERP) designed for patients with congenital duodenal obstruction

Recommendation	Definition of adherence	Percentage adherence (number adherent/number included)	Reason for non-adherence after ERP implementation
Antimicrobial prophylaxis			
A) Administer within 60 min prior to skin incision	A) Appropriate antibiotic prophylaxis within 60 min pre-incision	A) 16/21 (76%)	A) Not prescribed by surgeon
B) Discontinue within 24 h of surgery	B) Discontinuation within 24 h of surgery, unless ongoing treatment required	B) 21/21 (100%)	B) Not applicable
Perioperative communication			
Safe surgery checklist	Use of safe surgery checklist	21/21 (100%)	Not applicable
Surgical access			
Minimal surgical access	Supraumbilical access or laparoscopy	7/21 (33%)	Non- familiarity with minimally invasive access
Thermoregulation			
Maintain normothermia	Intraoperative body temperature between 36.5 and 37.5 ˚C	1/17 (6%)	At least one temp < 36.5˚C (n = 15) or > 37.5˚C (n = 4)
Fluid management			
Maintain tissue perfusion and prevent hypovolemia and fluid overload	“Goal-directed fluid resuscitation”	Unable to determine	No standard objective measurement tool available
Limit opioids			
A) Wound infiltration with bupivacaine	A) Perioperative wound infiltration with bupivacaine	A) 14/21 (67%)	A) Not administered
B) Dexmedetomidine infusion before extubation	B) Dexmedetomidine given as infusion while on ventilator	B) Unable to determine	B) Reason for no dexmedetomidine not documented
C) Regular acetaminophen postoperatively	C) Acetaminophen administered regularly	C) 21/21 (100%)	C) Not applicable
D) Clonidine as needed postoperatively	D) Clonidine administered if insufficient analgesia with acetaminophen alone	D) Unable to determine	D) Reason for no clonidine not documented
E) Lingual sucrose for minor procedures	E) Administer lingual sucrose prior to minor procedures	E) Unable to determine	E) Use of sucrose not documented
Optimal hemoglobin			
Restrictive transfusion practice	Transfusion only if HgB < 9 g/dL if no oxygen requirement and HgB < 11 g/dL if oxygen requirement	19/21 (90%)	Decision to transfuse based on clinical status
Urinary catheter			
Early removal of urinary catheter	Urinary catheter discontinued within 24 h after extubation	17/20 (85%)	Unknown (n = 2), concomitant anorectal malformation (n = 1)
Postoperative nutrition			
A) Early enteral feeding	A) First feed within 48 h after surgery	A) 21/21 (100%)	A) Not applicable
B) Breastmilk as first feed	B) Breastmilk used at initiation	B) 17/21 (81%)	B) Unknown (n = 2), breastfeeding not desired (n = 1), delayed maternal milk production (n = 1)
Parental involvement and counselling			
A) Early parental involvement	A) Ensure early parental involvement	A) Unable to determine	A) Not documented
B) Facilitate early breastfeeding	B) Breastmilk as main nutrition at discharge	B) 19/21 (90%)	B) Breastfeeding not desired (n = 2)
C) Provide written parental information	C) Provide written parenteral information	C) 21/21 (100%)	C) Not applicable

### Qualitative experiences with ERP implementation

Before implementation, the majority of the pediatric surgical colleagues were not familiar with the concept of ERAS. At the first meeting at site 1 where the ERP for CDO was presented to the pediatric surgical colleagues, there was significant resistance and skepticism towards the planned implementation. This was mainly rooted in the sum of suggested changes, particularly to change the standard transverse laparotomy to minimal access incision. Nevertheless, the idea of standardization of treatment based on the best available evidence was appreciated. The presence of a surgeon with broad experience in the development and implementation of ERAS in adult surgery was especially helpful in this phase of the project. The ERP team acknowledged that implementing minimally invasive surgery for a rare congenital gastrointestinal anomaly would be challenging and accepted that for this recommendation, compliance would initially be low. After several meetings, the protocol was widely accepted among the surgical colleagues.

In the following step, the protocol was presented to the nurses. They had previously expressed frustration due to variation in the postoperative management after CDO repair, and they instantly embraced the idea of standardization of care. They were especially enthusiastic about the nurse-driven feeding protocol, allowing the mothers to start early breastfeeding. According to the feeding protocol, aspiration of gastric residuals should only be done in case of nausea, vomiting or an unwell child. Weaning the nurses off routine aspiration of gastric residuals was demanding and required very close follow-up by the ERP team.

Early involvement of the parents was encouraged by the surgeons and the nurses. The parents were supported in early caretaking of their baby, exemplified by activities such as changing the nappy, administering feeds, and kangaroo care. The mothers were encouraged to breast-pump and were allowed to start breastfeeding the day after extubation.

After implementation at site 1, the ERP for CDO was presented at site 2. Initially, both physical and virtual meetings were arranged, and there was frequent contact between the ERP teams after implementation.

The ERP had immediate leadership approval and support at both sites. Members of the ERP teams were always available during the treatment of the included patients. Stakeholder feedback was encouraged and appreciated through regular meetings and close bedside follow-up during the treatment of patients with CDO after implementation. Experiences and challenges with the ERP were consecutively identified. Several adjustments of the protocol were necessary during the treatment of the first patients after implementation. Optimizing the feeding protocol was the main challenge, as it had to cover the variance in tolerance for feeding advancement in all patients. The nurses were an invaluable resource in adjusting and optimizing the feeding protocol. After the treatment of the first six patients, the feeding protocol was intuitive, easy-to-use, and well-functioning.

### Clinical outcome

ERP implementation resulted in several changes in both treatment and clinical outcome (Table 3). The use of supraumbilical and laparoscopic access and perioperative administration of dexmedetomidine increased. The use of TAFT decreased, and time to first enteral and breast feed were reduced. The incidence of postoperative complications and length of hospital stay remained unchanged.

**Table 3:** Clinical outcome after repair of congenital duodenal obstruction (CDO) in patients treated before and after implementation of an Enhanced Recovery Protocol (ERP)

	Before ERP implementation (2015–2020) *n* = 40	After ERP implementation (2022–2024) *n* = 21	*p*-value
Surgical access for CDO repair, n (%)			
Upper transverse laparotomy	39 (98)	14 (67)	0.003
Supraumbilical incision	1 (3)	5 (24)	
Laparoscopy	0 (0)	2 (10)	
Patients treated with dexmedetomidine perioperatively, n (%)	1 (3)	6 (29)	0.002
Patients treated with clonidine postoperatively, n (%)	5 (13)	6 (29)	0.121
Days with opioids after extubation, median (IQR)	1.0 (1.5)	0.0 (1.0)	0.260
Fluid balance 24 h from surgery (milliliters), median (IQR)	151 (159)	115 (102)	0.419
Patients treated with TAFT, n (%)	28 (70)	1 (5)*	0.000
Postoperative days with parenteral nutrition, median (IQR)	9.0 (6.0)	8.0 (4.5)	0.305
Postoperative days to first enteral feed, median (IQR)	2.0 (3.0)	1.0 (1.0)	0.000
Postoperative days to first breastfeed, median (IQR)	10.0 (5.0)	4.0 (2.0)	0.001
Complications, n (%)			
Clavien-Madadi grade ISSI without			
Antibiotics	1 (3)	2 (10)	0.146
Atelectasis	1 (4)	0 (0)	
TAFT-related	7 (18)	0 (0)	
Clavien-Madadi grade II			
SSI with antibiotics	7 (18)	2 (10)	0.791
CLABSI	1 (3)	1 (5)	
Sepsis of unknown origin	1 (3)	1 (5)	
Length of hospital stay (days), median (IQR)	14.0 (6.8)	13.0 (5.5)	0.461

^*^TAFT removed postoperative day one

*SSI* Surgical site infection, *TAFT* Transanastomotic feeding tube, *CLABSI* Central line-associated bloodstream infection

## Discussion

This is the first study to present an ERP specifically developed for CDO based on the ERAS guidelines for neonatal surgery. Unique for this study, is the presentation of the development of an ERP for CDO and the experiences with a nationwide 2-center implementation. Early results suggest that the ERP is feasible and safe.

The high ERP compliance rate of 80% was probably related to leadership approval and support, meticulous selection of the ERP team members, close follow-up and involvement of all stakeholders [28–30]. The ERP was well implemented at site 1 before it was presented to site 2, which made implementation at site 2 less complicated.

The correlation between ERP compliance and improved postoperative recovery is well described [31]. Still, changing clinical practice is challenging, even in the setting of well documented recommendations [32, 33]. Changing the standard upper transverse laparotomy to minimal access incision and to not routinely aspirate the gastric residuals generated skepticism among the pediatric surgical colleagues and nurses, hence, represented a barrier to successful implementation. Further, maintaining perioperative normothermia in neonates was very challenging. The recommendation of maintaining perioperative normothermia allowing no abnormal measurements is restrictive and difficult to adhere to, even with pre-warming of the operating room, use of heated operating table, underbody forced-air warmers, and continuous temperature monitoring [14, 34].

According to the ERAS guidelines for neonatal intestinal surgery, the use of regional analgesia is recommended to reduce the perioperative opioid requirement [9]. Compared to previous studies, the postoperative use of opioids in this study was very low, even in patients who had undergone a laparotomy [35, 36]. This suggests that regional anesthesia may not be necessary in neonates treated for CDO.

The use of an ERP for CDO has to date only been described in three publications [14–16]. Two of the protocols were specifically developed for CDO [15, 16]. In the first study, reduced length of hospital stay was found in 23 patients treated according to an ERP for CDO, focusing on early extubation and early initiation of enteral feeding compared to a control group (*n* = 26) [16]. However, this study had significant selection bias. Only patients with a prenatal diagnosis of CDO were treated according to the ERP, and the control group consisted of patients with postnatal diagnosis and therefore an increased risk of dehydration, infections, and electrolyte disorders. Recently, a randomized controlled trial demonstrated reduced time to first bowel movement, time to first postoperative breastfeeding, days with parenteral nutrition, and length of hospital stay in patients (*n* = 46) treated according to an ERP for CDO compared to a control group (*n* = 36) [15]. The ERP included measures such as early parental involvement, thermoregulation, fluid management and regional analgesia. None of these studies reported the process of protocol development and implementation, or protocol compliance. Additionally, the protocols were developed before the publication of the ERAS guidelines for neonatal intestinal surgery [9]. Consequently, some recommendations in the ERAS guidelines, such as to administrate appropriate antibiotic prophylaxis, to use a safe surgery check list, a restrictive transfusion practice, and to administrate regular acetaminophen, were not included. These recommendations have been included in the ERP for CDO in the current study. The third study reporting results after implementation of ERP for CDO did not use a protocol specifically designed for CDO, but reported the use of the ERAS guidelines for neonatal intestinal surgery in ten patients with duodenal and jejunoileal atresia, anorectal malformations, and segmental volvulus, respectively [14]. An overall protocol compliance of 73% was described, and measures with low compliance included discontinuation of antibiotic prophylaxis within 24 h after surgery, maintaining perioperative normothermia, administration of acetaminophen preoperatively, and initiation of enteral feeds within 48 h after surgery.

Early enteral feeding after neonatal intestinal surgery is safe and may reduce time with parenteral nutrition, reduce the risk for wound infections, and reduce length of hospital stay, without increasing the risk for complications [37–39]. In the current study, time to first enteral feeding was reduced after ERP implementation without an increase in postoperative complication rate. However, reduction of length of hospital stay could not be demonstrated. This may be due to the low number of included patients.

The World Health Organization recommend human milk over formula in all neonates [40]. Breastfeeding has been identified as a major concern among mothers of neonates treated for CDO, and a reduced success rate of breastfeeding in surgical neonates compared to non-surgical neonates has been described [21, 41, 42]. The nurse-driven feeding protocol in the current study encourages the mothers to start early breastfeeding and resulted in a significantly reduced time to first breast feed. This intervention may have several benefits beyond what can be objectively measured. Studies have demonstrated that the use of a TAFT can reduce time to full enteral feeds and time with parenteral nutrition after CDO repair [43]. However, medical equipment can act as a barrier against early breastfeeding [21, 44]. Hence, TAFT was not included in the ERP for CDO. This may have improved the success rate of breastfeeding after CDO repair.

Parents of neonates with a congenital anomaly requiring surgery have a significant need for information. In coherence with previous studies, the parents in the current study appreciated the information leaflet with structured written information (personal communication) [45, 46]. Prenatal counselling, web-based information and support groups are also reported to be appreciated by parents and will be an issue for future perspectives.

Patients with premature birth, major concomitant anomalies and chromosomal abnormalities have been excluded in previous studies of ERP for CDO [14–16]. In adult surgery, ERP is demonstrated to benefit both old patients and patients with significant comorbidity [47–49]. In the current study, half of the patients treated after ERP implementation were born prematurely and one third had concomitant anomalies. The exclusion criteria in this study are in line with previous publications regarding ERP for CDO [14, 16]. However, the evidence behind these exclusion criteria is weak, and treatment according to the ERP should be considered on an individual basis also in preterm infants and infants with concomitant anomalies. The feeding protocol in this study is designed for term and near-term neonates, and a dedicated feeding protocol for neonates with CDO with gestational age at birth < 32 weeks may be beneficial.

The main strength of this study is the description of the development and nationwide two-center implementation of an ERP specifically designed for CDO. The detailed description of the development of the protocol and the implementation process may be helpful for other centers planning to introduce an ERP for neonates with gastrointestinal conditions. Limitations include the low number of included patients. Further, the ERP was developed according to organizational standards in our hospitals and may therefore not be fully generalizable to other centers.

## Conclusions

We have presented the first ERP for CDO based on the ERAS guidelines for neonatal intestinal surgery and described our experiences with the development and implementation process. Lessons learned include the considerable need for preparation, information and close follow-up of all involved disciplines during implementation. Further, significant effort to achieve acceptance and enthusiasm for the profound changes of practice was necessary. For future perspectives and to improve power calculations of the benefits of the ERP for CDO, an initiative for multicenter implementation in the Nordic countries has been started.

## Data Availability

No datasets were generated or analysed during the current study.

## Abbreviations

CDO: Congenital duodenal obstruction
ERAS: Enhanced recovery after surgery
ERP: Enhanced recovery protocol
IQR: Interquartile range
SD: Standard deviation
